# Radiation-Resistant Microbes: Pioneers for Life Beyond Earth

**DOI:** 10.1007/s00284-026-04945-8

**Published:** 2026-05-16

**Authors:** Claudia Coleine, Radames JB Cordero

**Affiliations:** 1 Laboratorio de Biodiversidad y Funcionamiento Ecosistémico, Instituto de Recursos Naturales y Agrobiología de Sevilla (IRNAS), Consejo Superior de Investigaciones Científicas, Seville, Spain; 2 Department of Microbiology and Immunology, Johns Hopkins Bloomberg School of Public Health, Baltimore, MD 21205 USA

## Abstract

Radiation is one of the most pervasive environmental stressors that has profoundly influenced microbial evolution, driving the development of intricate survival strategies in extreme environments. This review examines the mechanisms underpinning microbial adaptation to various forms of radiation, including DNA repair systems, protective pigments, and antioxidant defenses. Emphasis is placed on the ecological niches occupied by radiation-resistant microbes, spanning from high-altitude deserts and deep subsurface ecosystems to space-exposed habitats. The insights gained not only enhance our understanding of microbial resilience but also hold significant implications for astrobiology and space exploration. By leveraging multi-omics approaches and experimental studies, research on radiation-resistant microbes has unveiled a broad range of potential applications in biotechnology, planetary protection, and the search for extraterrestrial life.

## Microbial Resilience to Radiation: A key to Life in Extreme Environments

Radiation is a critical environmental stressor that shapes microbial evolution, distribution, and ecological function. From intense ultraviolet (UV) radiation in high-altitude deserts to ionizing cosmic rays in space-exposed environments, microorganisms have evolved remarkable strategies to withstand and even thrive under extreme conditions [2, 6, 89]. In recent years, advancements in -omics technologies and space microbiology have provided unprecedented insights into the resilience of radiation-resistant microbes, highlighting their role in biogeochemical cycles and their potential applications in ecology, biotechnology and astrobiology [24, 25].

The urgency of studying microbial adaptation to radiation is driven by multiple converging factors. First, climate change is modifying microbial exposure to solar radiation, particularly in polar and arid ecosystems, through ozone variability, reduced cloud cover, and the loss of snow and ice that normally provide UV shielding [15, 67]. In polar regions, episodic ozone thinning and ice retreat increases biologically effective UV-B exposure at the surface, while in arid and high-altitude environments reduced atmospheric attenuation enhances UV stress on exposed microbial communities [63, 74, 82]. Because many of these communities operate at or near the soil surface and drive key carbon and nitrogen transformations, understanding their responses to changing radiation regimes is essential for predicting ecosystem resilience [26, 78]. Second, the rapid expansion of space exploration, with missions targeting Mars, Europa, Enceladus, and other celestial bodies, brings to the forefront crucial questions regarding microbial survivability in extraterrestrial environments [64, 79]. Research into extremophiles suggests that certain microbial life forms may be capable of enduring space-like environments, which has significant implications for planetary protection protocols and astrobiological investigations [47, 58, 69].

Microbial resistance to ionizing radiation not only informs our understanding of biological limits but also provides a valuable model for studying evolutionary mechanisms. Ancient and modern radiation-resistant species reveal how environments characterized by elevated exposure to ionizing radiation (e.g., gamma rays, X-rays, cosmic radiation) or biologically effective ultraviolet radiation (UV-B and UV-C) can act as selective pressures shaping microbial survival strategies [33, 56, 62, 80]. These adaptations may reflect early Earth conditions, offering a window into the origins of life and the potential for life to emerge under analogous extraterrestrial circumstances. A well-documented example is provided by endolithic microbial communities in Antarctica’s McMurdo Dry Valleys, where cyanobacteria, fungi, and heterotrophic bacteria colonize the interior of translucent rocks [40, 76]. These lithic substrates significantly attenuate UV radiation while permitting limited photosynthetically active radiation, enabling metabolic activity under extreme cold, desiccation, and radiation stress. Metagenomic analyses have revealed an enrichment of DNA repair pathways, antioxidant systems, and stress-response genes in these communities [26, 105]. These features enable microbial survival under extreme conditions and make these environments useful analogues for potential microbial habitats in the Martian subsurface. A second example comes from deep subsurface radioactive ecosystems, such as uranium-rich fracture waters and ancient crystalline rock formations, where microbial life persists under chronic exposure to ionizing radiation and extreme energy limitation [94]. In these environments, microorganisms can use hydrogen produced by radiolysis, namely the breakdown of water by radiation, as an energy source, together with sulfate or metal reduction pathways, to sustain metabolism independently of sunlight. These processes make such systems relevant analogues for potential life in the subsurface oceans of icy moons, such as Europa and Enceladus, where radiolysis at ice–rock interfaces may similarly provide energy for microbial ecosystems [21, 104].These findings challenge the traditional Earth-centric perspective of habitability and broaden the criteria used in the search for extraterrestrial biosignatures [39].

Additionally, microbial extremophiles have significant applications in biotechnology. Their unique DNA repair mechanisms and protective biomolecules, such as melanin in fungi and carotenoids in bacteria, are of growing interest for developing radiation-resistant biomaterials, improving cancer radiotherapies, and enhancing bioremediation of radioactive waste [35, 97]. Recent studies have unraveled highly efficient DNA repair systems that could inspire synthetic biology applications, including the engineering of radiation-resistant microbial strains, the development of robust genetic circuits for extreme environments, and the design of DNA repair-based tools for genome stability in biotechnological and medical applications [54]. Furthermore, microbial biofilms have been shown to provide collective protection against radiation, an adaptation that could be harnessed for space-based biotechnologies [100].

Early studies on radiation-resistant microorganisms date back to the mid-20th century, with the discovery of highly resistant species such as *Deinococcus radiodurans*, which revealed the existence of robust DNA repair systems capable of maintaining genome integrity under extreme radiation exposure. Since then, research has progressively expanded from single-organism studies to a broader understanding of radiation resistance as a multifactorial trait involving coordinated DNA repair, antioxidant defenses, metabolic regulation, and ecological interactions. Recent advances in genomics, metagenomics, and space exposure experiments have further deepened our understanding of how microbial communities respond to radiation across diverse environments. Despite these advances, several key questions remain unresolved. It is still unclear to what extent radiation resistance mechanisms are evolutionarily conserved across taxa, or whether they represent convergent adaptations to multiple environmental stressors. In addition, the regulatory networks that coordinate responses to combined stresses such as radiation, desiccation, and nutrient limitation remain poorly understood. Finally, the development of quantitative and predictive models to assess microbial survival under extraterrestrial conditions is still in its early stages, limiting our ability to extrapolate laboratory findings to planetary environments.

Rather than providing an exhaustive catalogue of all known radiation-resistant taxa, this review focuses on a selection of well-characterized organisms and environments to illustrate the principal survival strategies and molecular mechanisms underlying radiation tolerance. While this review focuses primarily on organism-level adaptations, ecological and community-level processes are included when supported by strong field evidence and when relevant to ecosystem functioning, biogeochemical cycling, or applied contexts such as bioremediation and space environments. This targeted approach aims to integrate mechanistic insights with ecological relevance, without attempting a comprehensive survey of all radiation-impacted microbial communities.

## Types of Radiation and their Biological Impact on Microorganisms

Microbes thriving in high-radiation environments must endure exposure to a variety of radiation types, including cosmic rays, UV radiation, gamma rays, and X-rays, along with additional stressors such as microgravity, desiccation, and vacuum conditions. These forms of radiation differ in their energy levels, penetration abilities, and biological impact, requiring specialized microbial adaptations for survival.

UV radiation, primarily emitted by the Sun, is a significant challenge for microbes living on planetary surfaces with little or no atmospheric shielding. As a form of non-ionizing radiation, UV does not directly ionize atoms but can still cause extensive cellular damage. UV-A (315–400 nm) penetrates microbial cells and induces oxidative stress, leading to protein and lipid damage. UV-B (280–315 nm) is particularly harmful to DNA, as it promotes thymine dimer formation, resulting in mutations that can compromise genetic stability. The most damaging of all, UV-C (200–280 nm), is effectively filtered by Earth’s atmosphere but reaches planetary surfaces lacking atmospheric protection. On Earth, biologically effective UV-B radiation increases substantially with altitude, with surface UV doses at high-altitude or polar sites exceeding those at sea level by several-fold, particularly under ozone-thinning conditions [14]. In contrast, on Mars and the Moon, the absence or extreme thinning of an atmosphere allows intense UV fluxes, resulting in UV radiation levels that are orders of magnitude higher than those experienced by surface ecosystems on Earth. Particularly, on Mars the thin CO₂-dominated atmosphere provides little attenuation in the 200–400 nm range, allowing both UV-B and UV-C radiation to reach the surface. Radiative transfer models and spacecraft observations indicate that the Martian surface is exposed to continuous, broadband UV fluxes across this spectral range, resulting in biologically effective UV doses that are orders of magnitude higher than those experienced by terrestrial surface ecosystems (Vázquez & Hanslmeier et al., [19, 96]). Consequently, UV radiation represents one of the primary limiting factors for microbial survival on exposed extraterrestrial surfaces [22, 36]. While the initial focus often lies on the high-energy impacts of ionizing radiation, non-ionizing radiation can also be significantly deleterious to microorganisms, with effects heavily dependent on the frequency and dose of exposure. Excessive absorption of non-ionizing radiation occurs when cells absorb more radiant energy than they can dissipate, resulting in an increase in cellular temperature. This overheating can indirectly promote cellular dehydration by enhancing water loss and destabilizing macromolecular structures, but dehydration itself is a secondary consequence rather than the primary stressor. In this context, thermal damage and protein denaturation arise from radiation-induced heating, whereas desiccation represents an associated but distinct physiological challenge [48]. Notably, the degree of radiation absorption can vary significantly among microorganisms. For instance, darkly pigmented yeast are known to absorb electromagnetic radiation more efficiently than their lighter counterparts, leading to greater heat capture under solar exposure [16, 27, 52, 113]. This pigment-mediated thermal absorption presents an ecological trade-off: enhanced solar warming confers a growth advantage in cold environments but becomes a liability at warmer ambient temperatures, where excessive heating can impair viability. This pigment-mediated heat absorption phenomena offers a leading explanation for the observed prevalence of darkly pigmented yeast species in high-latitude, cold environments.

Ionizing radiation encompasses several components that differ markedly in origin, energy, and environmental prevalence [4, 36]. On Earth, background ionizing radiation is relatively low (approximately 2–3 mSv yr⁻^1^), largely due to shielding by the atmosphere and magnetosphere [36]. This radiation mainly originates from terrestrial radionuclide decay and from secondary cosmic-ray particles generated in the upper atmosphere. At high altitudes and polar regions this shielding is reduced, leading to increased exposure, but still far below levels encountered in extraterrestrial settings. Beyond Earth, ionizing radiation is dominated by galactic cosmic rays (GCRs) and solar energetic particles (SEPs), which include high-energy protons, alpha particles, and heavy ions. On the Moon, the absence of both atmosphere and global magnetic field results in continuous exposure to GCRs and episodic SEP events, yielding cumulative surface doses on the order of hundreds of mGy yr⁻^1^ to >1 Gy yr⁻^1^, depending on solar activity and depth below the regolith. On Mars, the thin atmosphere (~1% of Earth’s surface pressure) provides only partial attenuation; measurements from the Mars Science Laboratory indicate surface dose rates of ~0.2–0.3 mGy day⁻^1^, corresponding to ~70–80 mGy yr⁻^1^, with additional contribution from secondary particles generated in the regolith [36]. In contrast, subsurface environments, both on Mars and on icy moons such as Europa and Enceladus, experience steep radiation gradients, where shielding by rock, ice, or regolith can reduce biologically relevant doses by orders of magnitude. Importantly, while UV radiation primarily affects surface-exposed cells, ionizing cosmic radiation penetrates deeply into materials and biological aggregates, producing dense ionization tracks, clustered DNA damage, and protein oxidation that are substantially more difficult to repair.

## Ecological Niches of Radiation-Resistant Microbes

### High-Altitude and Desert Environments

High-altitude and desert environments represent some of the most extreme ecosystems on Earth, characterized by intense UV radiation, desiccation, and limited nutrient availability. Among these, the Atacama Desert and the Antarctic Dry Valleys stand out as two of the most hyper-arid and radiation-intense environments, offering unique insights into microbial survival under extreme conditions. In the Atacama Desert, surface UV Index values during the austral summer are expected to exceed 11 across the entire desert, and annual UV-B doses range from ~3.5 kWh m⁻^2^ in coastal areas to ~5 kWh m⁻^2^ on the Andean plateau, values that are approximately 40% higher than those reported for other major hot deserts such as northern Africa [27, 113]. Similarly, Antarctic Dry Valley regions experience episodic extreme UV exposure during austral spring due to ozone thinning, placing them at the upper end of biologically relevant UV radiation on Earth [12]. Even the most extreme environments on Earth receive lower UV radiation than the surface of Mars. Due to the absence of an ozone layer, Mars is continuously exposed to high levels of broadband UV radiation, including UV-C. The Atacama Desert in northern Chile is the driest non-polar desert in the world, with some regions receiving virtually no rainfall for centuries [3, 87]. Despite these inhospitable conditions, recent metagenomic and microbial ecology studies have revealed the presence of specialized microbial communities adapted to extreme UV exposure and desiccation, including cyanobacteria such as *Chroococcidiopsis*, Actinobacteria, and melanized fungi. Research has shown that microbial life in the Atacama desert is primarily found below the soil surface or within salt crusts, where organisms can access minimal water and are shielded from radiation [99, 118]. These microbes can survive prolonged dormancy during dry periods and reactivate when minimal moisture becomes available. Moreover, hypolithic microbial communities, those living underneath translucent quartz rocks, are typically dominated by cyanobacteria such as *Chroococcidiopsis*, along with heterotrophic bacteria and fungi that persist under reduced radiation and increased moisture availability [64, 106]. The McMurdo Dry Valleys in Antarctica are among the coldest and driest environments on Earth, with conditions comparable to those of the Atacama Desert but further intensified by sub-zero temperatures and strong katabatic winds. Here, microbial life is mainly found in soils and endolithic communities, organisms that colonize the interior of porous rocks, where they are insulated from temperature fluctuations and radiation [40]. Importantly, the prevalence of endolithic microbial communities does not merely reflect protection from temperature extremes but represents a functional strategy to mitigate radiation stress. In Antarctic cryptoendolithic systems, melanized black fungi typically colonize the outermost millimeters of the rock substrate, forming a pigmented biological layer that strongly attenuates incoming damaging radiation [23, 112]. This melanized layer acts as an effective radiative filter, significantly reducing radiation penetration into the rock interior and thereby shielding underlying phototrophic and heterotrophic microorganisms (e.g., algae and bacteria) from direct irradiation. As a result, radiation resistance in these systems emerges not only from individual cellular adaptations, but from spatial organization and community structure, where melanized fungi function as ecosystem engineers that enable the persistence of radiation-sensitive taxa in otherwise lethal surface conditions [40]. This stratified organization highlights how community-level interactions and functional partitioning can enhance survival in radiation-intense environments, providing a terrestrial analogue for potential subsurface or endolithic life on Mars

In this context, community-level interactions play a crucial role in shaping the functional resilience of radiation-resistant microbiomes. In extreme environments microorganisms rarely exist in isolation. In such settings, microbes commonly organize into structured consortia, often forming biofilms or spatially organized aggregates that modify local physicochemical conditions and reduce individual exposure to radiation. Biofilm matrices and extracellular polymeric substances can partially attenuate incoming radiation, limit the diffusion of reactive oxygen species, and create microenvironments with reduced oxidative stress, thereby enhancing collective survival. At the community level, radiation resistance is further reinforced by functional redundancy, whereby multiple taxa perform similar metabolic functions. This redundancy allows essential processes such as carbon turnover, nitrogen transformations, or sulfur cycling to persist even if radiation selectively damages or eliminates sensitive members of the community. In radiation-impacted or radioactive environments, microbial consortia involved in anaerobic nitrogen cycling, methanogenesis, or metal reduction have been shown to remain metabolically active, sustaining energy flow and biogeochemical functioning under chronic stress [101, 120]. In addition, syntrophic interactions within microbial communities can mitigate radiation-induced limitations by coupling energetically unfavorable reactions to complementary metabolisms, increasing overall system stability. In environments contaminated by radionuclides, community-level processes such as microbial uranium reduction or the binding of cesium and other radionuclides to extracellular polymeric substances can influence element mobility and bioavailability, highlighting the role of microbial assemblages in natural attenuation and bioremediation [86, 119]. For example, syntrophic interactions between hydrogen-producing and hydrogen-consuming microorganisms can stabilize redox conditions and sustain metabolism under radiation stress. In deep subsurface systems, fermentative or radiolysis-driven processes generate H₂, which is then utilized by sulfate-reducing bacteria or methanogenic archaea, enabling energy flow in low-energy environments.

While the Atacama Desert and the Antarctic Dry Valleys have been extensively studied as Mars analogs, recent discoveries have expanded our understanding of radiation-resistant microbes in other extreme locations. These environments, ranging from the Tibetan Plateau and Mount Everest to the Lut Desert in Iran and the Taklamakan Desert in China, provide further insights into how life adapts to high-radiation conditions. These habitats provide valuable insights into the physiological limits and adaptive traits required for microbial survival under the combined stressors of intense UV radiation, low atmospheric pressure, and extreme desiccation that characterize the Martian surface [107]. Recent studies have uncovered novel radiation-resistant bacterial species, including *Knollia* sp. nov. S7-12T, isolated from the North Slope of Mount Everest at over 6,500 meters [102]. Similarly, two novel bacteria isolated from the high-altitude permafrost of Mount Everest, have demonstrated extreme radiation tolerance, likely due to their highly efficient DNA repair mechanisms and production of carotenoid pigments [53, 115]. The discovery of these extremophiles in the Himalayas underscores the potential for microbial survival in the stratosphere and beyond. The Taklamakan Desert, China’s largest and highly radiation-exposed desert, harbors unexpectedly abundant microbial life. Recent studies found that over 30% of culturable bacteria from its deep sands exhibit radiation resistance, a much higher proportion than previously estimated [53, 115]. Among these, Actinobacteria and *Deinococcus* species thrive in subsurface niches, where they are shielded from UV radiation yet remain exposed to background ionizing radiation from cosmic and terrestrial sources. Beyond studies on isolated high-altitude strains, several investigations have directly examined atmospheric and stratospheric microbial communities, demonstrating that viable microorganisms can persist, disperse, and potentially remain metabolically active under low pressure, intense UV radiation, and oxidative stress conditions analogous to those encountered in the upper atmosphere and near-space environments [92],Santl-Temkiv et al., [85].

One of the most unexpected findings in desert areas has been the presence of radiation-resistant fungi, such as species within the genus *Exophiala*, which are commonly associated with radiation-rich environments like nuclear reactor sites. In these settings, melanized fungi have been shown to persist under chronic exposure to ionizing radiation, where melanization and efficient oxidative stress responses are thought to contribute to cellular protection and long-term survival [31, 117]. The repeated occurrence of similar fungal taxa in both anthropogenic radioactive sites and naturally extreme environments suggests that fungal extremophiles possess adaptive traits enabling tolerance to sustained radiation exposure, highlighting their potential ecological relevance in radiation-impacted ecosystems [5, 24].

### Deep Subsurface Ecosystems and Naturally Radioactive Environments

Deep subsurface ecosystems and naturally radioactive environments are among the most isolated and extreme habitats on Earth, where microbial life is subjected to persistent ionizing radiation, high pressure, and severe energy limitations. These environments, which include kilometer-deep rock formations, uranium-rich groundwater systems, and deep-sea hydrothermal vents, provide unique insights into how life can persist under chronic exposure to radiation sources such as gamma rays, alpha and beta radiation from radioactive decay, and secondary cosmic rays penetrating Earth’s crust [49, 110]. Importantly, these ecosystems demonstrate that radiation can act as a persistent selective pressure and, in some cases, an indirect energy source, through radiolysis-driven production of hydrogen and other reduced compounds that sustain microbial metabolism. Radiolysis-driven metabolism is based on the dissociation of water molecules by ionizing radiation, producing molecular hydrogen (H₂), oxidants, and reactive intermediates. The generated H₂ can serve as an electron donor for chemolithoautotrophic microorganisms, supporting metabolic pathways such as sulfate reduction, methanogenesis, and metal reduction in the absence of sunlight. This process has been documented in deep subsurface environments, where *Desulforudis* relies on radiolytically produced hydrogen to sustain an independent ecosystem [57]. Similar mechanisms have been proposed for deep crystalline rocks and subsurface aquifers, where radiolysis provides a continuous, low-level energy source that supports long-term microbial persistence under extreme energy limitation.

These observations highlight how long-term exposure to ionizing radiation shapes both resistance mechanisms and ecosystem functioning, offering analogs for potential subsurface life on other planetary bodies.

Microbial life in the deep biosphere has been detected at depths exceeding 2.5 kilometers below the surface. In these environments, organisms are exposed to persistent background ionizing radiation from the decay of naturally occurring radionuclides such as uranium, thorium, and potassium. Some microbes can also use hydrogen generated by radiolysis as an energy source. Although absolute dose rates in most deep terrestrial subsurface settings are generally lower than surface acute exposures, the chronic, long-term presence of ionizing radiation imposes a continuous selective pressure that necessitates efficient DNA repair and oxidative stress mitigation. In South Africa’s Witwatersrand Basin, a system of deep fractures within gold mines has revealed hydrogenotrophic sulfate-reducing bacteria, including *Ca. Desulforudis audaxviator*, capable of surviving independently from sunlight and organic carbon sources [20]. These microbes rely on radiolytic hydrogen, a product of radioactive decay, as a primary energy source, demonstrating that radiation can drive deep biosphere ecosystems [71]. This finding implies that radiation is not solely a stressor but can act as a long-term energy driver in isolated environments, supporting microbial survival over geological timescales [77]. Importantly, this process operates under low but persistent radiation fluxes, where energy availability and damage repair remain balanced, beyond certain thresholds, excessive radiation would overwhelm cellular repair mechanisms and limit viability. These insights extend the potential habitability of subsurface environments on Earth and other planetary bodies, such as Mars or icy moons, where radiolysis may similarly sustain life shielded from surface radiation. Similarly, in naturally radioactive groundwater systems, such as those in Finland’s deep boreholes and Canada’s uranium-rich Cigar Lake, microbial communities are regularly exposed to alpha and beta radiation emitted from decaying uranium and radon [38, 81]. Recent studies indicate that microbial life in deep subsurface environments can persist over geological timescales through ultra-slow metabolism, dormancy, and efficient maintenance and repair processes under chronic low-dose ionizing radiation [98]. Evidence for this persistence includes isotopic disequilibria, genomic stasis, and extremely long generation times, ranging from centuries to millennia. These systems suggest that sustained habitability under radiation exposure is possible when combined with shielding by rock matrices, radiolysis-driven energy sources, and community-level metabolic interactions. Although such environments provide useful analogues for potential subsurface habitats on Mars or icy moons, differences in radiation fluxes and geochemical conditions limit direct extrapolation. Yet recent research has demonstrated the potential of certain microbial populations to actively contribute to radionuclide bioremediation. Species such as *Geobacter* and *Desulfovibrio* are particularly effective in reducing soluble forms of uranium and other radioactive elements to insoluble states, thereby decreasing their mobility and limiting the risk of groundwater contamination [55]. Field-based studies at radioactive waste repositories have revealed diverse communities dominated by denitrifying, iron-reducing, and sulfate-reducing bacteria, including members of *Pseudomonas*, *Thermomonas*, *Rhizobium*, *Brevundimonas*, *Acidovorax*, *Bacillus*, and *Paenibacillus* [83]. In these systems, nitrate often represents a primary geochemical driver shaping community composition, with denitrification acting as a prerequisite for subsequent uranium and radionuclide immobilization under reducing conditions. This community-level organization enhances ecosystem stability, limits radionuclide mobility, and underpins the effectiveness of in situ bioremediation strategies at nuclear-contaminated sites, highlighting the importance of microbial ecology, rather than single-species resistance, in radiation-associated biotechnological applications. These microbial processes not only mitigate the environmental impact of radioactive waste but also offer promising avenues for the development of sustainable bioremediation strategies in nuclear-contaminated sites. Beyond deep subsurface ecosystems, certain microbial communities thrive in areas of naturally elevated radioactivity. The Chernobyl Exclusion Zone serves as a prime example of an ecosystem where life has adapted to chronic radiation exposure. Studies have identified melanized fungi, such as *Cladosporium sphaerospermum and other dematiaceous fungi* colonize highly radioactive structures, utilizing melanin to shield themselves from ionizing radiation (Dighton et al., 2008). Fungal species isolated from the exclusion zone exhibit radiotropism, that is, attraction to ionizing radiation sources [133]. Studies on melanin-producing fungi showed that melanized cells exhibit enhanced growth and increased metabolic activity when exposed to ionizing radiation [31]. This process, termed radiosynthesis, describes the apparent conversion of radiation energy into a form that fungal cells can use to support growth. While the detailed molecular mechanisms of radiosynthesis are still under investigation, it is understood that it involve the semiconductor and redox properties of melanin. Ionizing radiation alters the polymer's electronic structure and oxidation state, enabling electron-transfer reactions that can oxidize NADH and generate reductants accessible to cellular metabolism [17]. In this context, radiation is not merely a stressor but appears to be coupled to cellular metabolism, likely through changes in the electrical and redox properties of melanin upon irradiation. Although the precise molecular pathways linking melanin-mediated energy absorption to cellular metabolism remain incompletely understood, these observations indicate a form of radiation-supported metabolism rather than passive survival alone.

These findings support the idea that radiation can act as an indirect energy source for life, for example through radiolysis-driven hydrogen production and the generation of secondary electrons that fuel biochemical processes (Stelmach et al., 2018). This expands current models of habitability in radiation-rich terrestrial and extraterrestrial subsurface environments.

Microbial life in deep-sea hydrothermal vents may also experience ionizing radiation, primarily from radioactive isotopes naturally present in vent fluids and the surrounding crust. These environments harbor extremophiles such as *Thermococcus gammatolerans*, a deep-sea archaeon isolated from hydrothermal sediments, which has demonstrated exceptional resistance to gamma radiation, tolerating doses exceeding 30,000 Gy [49]. Genome and proteome analyses indicate that this exceptional radioresistance is not driven by an expanded repertoire of canonical DNA repair genes, but rather by a combination of highly efficient DNA repair kinetics, robust protein protection systems, and cellular components that remain stable under extreme physicochemical stress [8, 109]. In particular, enhanced protein stability, detoxification pathways, and redox homeostasis appear central to limiting radiation-induced damage at high temperatures. At the ecosystem level, these traits support the persistence of microbial communities in hydrothermal systems under combined stresses such as heat, pressure, and radiation. This, in turn, helps maintain functional members of the community and contributes to the stability of vent-associated biogeochemical processes.

Together, these extreme environments, from hyper-arid deserts to deep subsurface systems, highlight the capacity of microbial life to persist under chronic radiation, desiccation, and energy limitation. Adaptations such as desiccation tolerance, rapid metabolic recovery, and the use of radiolytically derived energy sources support survival in radiation-rich habitats [29]. These findings have direct astrobiological relevance, suggesting that similar microbial ecosystems could exist beyond Earth in protected niches such as the Martian subsurface or the ice-covered oceans of icy moons. Natural analogues like the Oklo nuclear reactor further illustrate the potential for life under long-term ionizing radiation [30].

## Microbial Strategies for Radiation Resistance

**Fig. 1:**
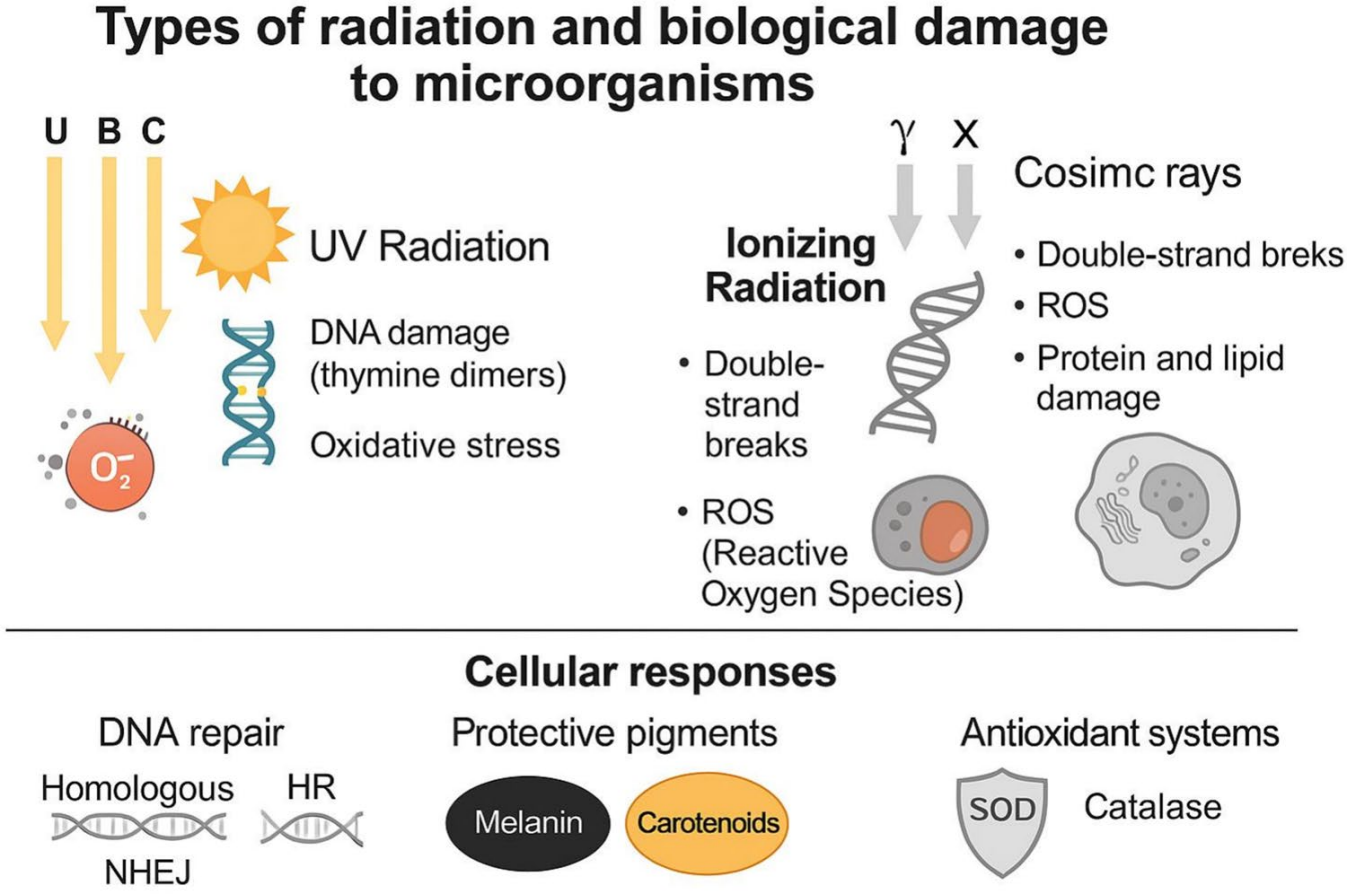
Conceptual overview of radiation-resistant microbes and their relevance to the total environment. The figure summarizes the main sources of environmental radiation and microbial adaptations enabling survival. Key cellular responses include DNA repair pathways; HR, homologous recombination; NHEJ, non-homologous end joining; SOD, superoxide dismutase

Microorganisms inhabiting high-radiation environments have evolved a remarkable set of physiological and biochemical mechanisms to endure extreme conditions (Fig. 1). Chief among these adaptations is an array of DNA repair mechanisms, which enable cells to detect and repair damage caused by ionizing and ultraviolet radiation. The bacterium *D. radiodurans*, one of the most radiation-resistant organisms known, exemplifies this ability through highly efficient DNA repair processes, primarily based on the Extended Synthesis-Dependent Strand Annealing (ESDSA) pathway [108]. Earlier work shows that it can tolerate acute exposures exceeding 10,000 Gy. This resilience is supported by a tightly packed toroidal genome structure that minimizes DNA fragmentation and enables highly efficient DNA repair mechanisms, including ESDSA and robust antioxidant defenses [73, 90].

Another effective strategy to cope with radiation-induced DNA damage, particularly widespread among Archaea, is the presence of polyploid genomes. Multiple genome copies provide redundancy that facilitates efficient DNA repair through homologous recombination and compensates for extensive damage caused by both ionizing and non-ionizing radiation. Polyploidy has been documented in several archaeal lineages, including halophilic and thermophilic species, and has been shown to enhance survival following irradiation by allowing accurate genome reassembly even when individual chromosome copies are severely fragmented (Soppa & Soppa, 2019).

**Fig. 2:**
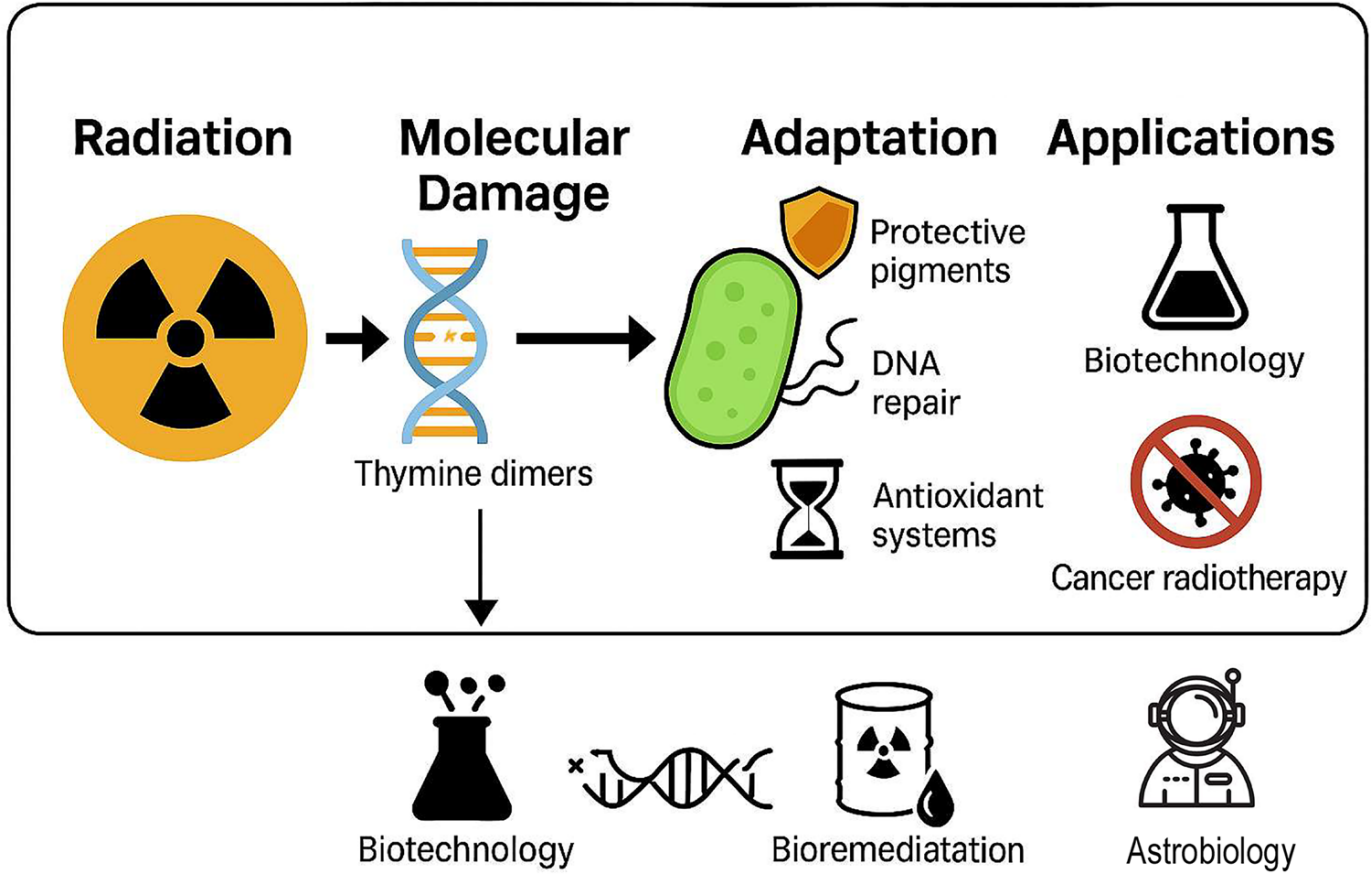
Schematic representation of the potential applications of radio-resistant microorganisms. The figure illustrates, in a graphical manner, the wide range of possible uses of extremophilic microorganisms with high resistance to ionizing radiation

Yet, many microbes employ protective pigments and biomolecules to mitigate radiation damage. Melanin, a complex biopolymer found in numerous fungi and bacteria, plays a critical role in shielding cells from radiation by absorbing harmful wavelengths and dissipating energy harmlessly. Its radioprotective function operates through two main mechanisms. First, melanin acts as a physical shield by attenuating ionizing and non-ionizing radiation, absorbing harmful wavelengths and dissipating the energy as harmless heat. This reduces the direct impact of radiation on cellular structures. Second, melanin functions as a biochemical antioxidant, scavenging reactive oxygen species (ROS) generated during irradiation and thereby preventing oxidative damage to DNA, proteins, and lipids [1, 7, 32]. Within melanzed eukaryotes, fungi have emerged as highly resistant organisms [32]. Recently, large-scale screening of melanized fungi has identified species from the genera *Cryomyces* and *Friedmanniomyces* (Dothideomycetes, Ascomycota) as among the most radiation-tolerant black fungi tested to date [6]. These species maintain viability even at doses up to 50 kGy, highlighting their potential for space biology and radioprotection research. The basidiomycetous yeast *C. neoformans* and the filamentous fungus *Paecilomyces variotii*, for example, accumulate melanin in their cell wall, which has been shown to enhance their survival following exposure to cell-damaging radiation [50, 60]. In laboratory studies, melanized fungal cells exhibit greater resistance to ionizing radiation compared to non-melanized mutants, highlighting the protective function of this pigment Fig. 2.

In addition to melanin, carotenoid pigments represent a major and widespread radioprotective strategy across diverse microbial taxa. Carotenoids are particularly abundant in microorganisms inhabiting hypersaline environments, where intense solar radiation and high salinity co-occur. In these systems, carotenoids act both as photoprotective compounds by absorbing excess visible and UV radiation and as potent antioxidants that quench singlet oxygen and other reactive oxygen species generated during irradiation. For instance, the extremophilic bacterium *Sphingomonas desiccabilis*, produce carotenoid pigments, which not only serve as photoprotectants but also function as antioxidants, neutralizing reactive oxygen species generated by radiation exposure [41]. The dominance of carotenoid-rich microorganisms in hypersaline habitats underscores the importance of these pigments in mitigating radiation-induced oxidative stress under extreme environmental conditions.

Many radiation-resistant microbes, including *Rubrobacter radiotolerans* and *Deinococcus spp.*, possess elevated levels of superoxide dismutases, catalases, and peroxidases, enzymes that scavenge free radicals and limit oxidative stress. Research on *D. radiodurans* has revealed a unique intracellular environment rich in manganese antioxidants, which help protect cellular macromolecules from oxidation while preserving protein functionality [34]. This antioxidant system is so effective that synthetic versions are now being explored for radioprotective applications in medicine and biotechnology.

Bacterial species such as *Bacillus subtilis* and *Pseudomonas aeruginosa* form dense biofilms that enhance collective resistance to radiation, with cells in deeper layers benefiting from the protective shield of outer layers [45, 103]. Multicellular organization, such as the formation of microbial aggregates, plays a key role in enhancing resistance to radiation. Experiments on the International Space Station (ISS) have shown that such aggregates survive prolonged exposure to space vacuum and cosmic radiation far better than isolated cells [46, 59, 75].

### Space and Extra-Terrestrial Conditions Exposed Experiments

Despite these challenges, numerous experiments aboard the International Space Station (ISS), lunar missions, and Mars simulation platforms have demonstrated that certain microorganisms can persist, adapt, and even repair radiation-induced damage under such conditions.

The ESA’s EXPOSE programme comprised three independent exposure platforms (EXPOSE-E, EXPOSE-R, and EXPOSE-R2) deployed on the exterior of the ISS to investigate the survival of biological systems under space conditions. Within this framework, the BIOMEX (BIOlogy and Mars EXperiment) was conducted on the EXPOSE-R2 platform, with the primary objective of assessing the stability of biosignatures, such as pigments and cellular components, when exposed to space and Mars-analog mineral environments [37]. In addition to biosignature stability, BIOMEX provides insights into the viability of a range of microorganisms and biomolecules exposed to space and Mars-like conditions, offering valuable data toward understanding the limits of life and the potential for interplanetary transfer [37]. For instance, the cryptoendolithic black fungus *Cryomyces antarcticus*, isolated from Antarctic rock habitats, maintained survival, preserved DNA integrity and ultrastructural stability after prolonged exposure to space and simulated Mars-like conditions, including vacuum, UV radiation and temperature extremes [70]. Similarly, biofilms formed by desert strains of the cyanobacterium *Chroococcidiopsis* showed higher survival than planktonic cells when exposed outside the ISS under low Earth orbit conditions [13]. These experiments included exposure to space vacuum and Mars-analog UV radiation (200–400 nm, total fluence ~4.6–4.9 × 10^2^ kJ/m^2^). The results suggest that biofilm structure and extracellular polymeric substances enhance tolerance to prolonged space and Mars-like radiation. Together, results from EXPOSE-based experiments demonstrate that selected microorganisms can tolerate prolonged exposure to space-relevant stressors, while highlighting the importance of experimental design and shielding conditions in determining survival outcomes.

Within the framework of the TANPOPO mission aboard the ISS, cell aggregates of *D. radiodurans* were exposed on the exterior of the ISS for up to three years, surviving cumulative cosmic radiation and UV environment of low Earth orbit and demonstrating a remarkable capacity for DNA repair following prolonged space exposure [59]. Related experiments further showed that *D. radiodurans* cells and *Bacillus pumilus* spores could withstand extended exposure to Mars-like UV radiation conditions (approximately 1.5 W m⁻^2^ UV-C for over one year), highlighting the potential for microbial persistence under unshielded extraterrestrial radiation environments (Yamagishi et al., 2021).

*Bacillus pumilus* SAFR-032, originally isolated from spacecraft assembly facilities, exhibits high resistance to UV radiation (e.g., 200 J/m^2^ UV-C) and desiccation, enabling persistence on spacecraft surfaces [95]. Comparative studies show that *B. pumilus* displays significantly higher UV resistance than standard *Bacillus subtilis* biodosimetry strains, tolerating doses exceeding those typically used for spacecraft sterilization [51]. This resistance is largely attributed to intrinsic spore properties, including small acid-soluble spore proteins (SASPs) that protect DNA, and structural features of the spore coat that limit UV penetration [68, 91]. The persistence of *B. pumilus* in cleanroom environments, together with its demonstrated survival under space exposure and high-altitude conditions, highlights its relevance for planetary protection [61, 93].

Inside the ISS, *Cutibacterium* species have been found thriving in astronaut habitats, demonstrating an ability to form protective biofilms that shield against radiation and environmental stressors [116]. Recent large-scale, culture-independent surveys of the ISS microbiome further show that microbial community composition and metabolic profiles are strongly shaped by module usage and built-environment constraints, with human-associated taxa dominating this highly industrialized and isolated habitat [84].

Further studies have investigated microbial survivability under extraterrestrial surface and near-surface conditions, where organisms are exposed to vacuum, large temperature fluctuations, and intense radiation fields [114]. A recurring outcome is that even minimal shielding, by dust, regolith grains, or UV-attenuating substrates, can markedly alter microbial survival. Under simulated Martian conditions, Fe-sulfate brines (Fe₂(SO₄)₃) were shown to attenuate harmful UV radiation and enhance the survival of *B. subtilis* spores even at low concentrations, whereas MnSO₄ and MgSO₄ offered limited UV protection; importantly, the strong acidity of Fe-sulfate brines imposed an additional selective constraint on non–spore-forming cells [43]. Optical transmission measurements through Mars-analog regolith and rocks show that UV attenuation strongly depends on the material [44]. Some substrates act as effective UV “quenchers,” significantly reducing radiation penetration. Modeling further suggests that biologically relevant UV reduction can occur at sub-millimeter depths, with protection in some cases provided by a single grain of simulant. However, shielding effects are radiation-type dependent: under ionizing radiation, spores covered by artificial Martian regolith were reported to become more sensitive to X-rays, consistent with regolith–radiation interactions generating secondary electrons and reactive species [65].

Supporting the protective role of melanin in space, a recent study demonstrated that fungal melanin protects yeast against spaceflight stressors. In this study, melanized and non-melanized forms of *C. neoformans* were sent on a 29-day round trip to the interior of the ISS, and the melanized cells survived approximately 50% more than the non-melanized cells [28]. Extending these cellular-level findings to biomaterials, biocomposites of fungal melanin embedded in poly(lactic acid) (PLA-FunMel) were exposed on the exterior of the ISS and exhibited reduced mass loss and structural deformations relative to non-melanized PLA controls. This positions fungal melanin as a candidate radioprotective additive for spacecraft materials. 

## Concluding Remarks and Future Perspectives: Implications for Astrobiology and Space Exploration

The study of radiation-resistant microorganisms has significantly advanced our understanding of life under extreme conditions. Recent research highlights a shift from single-factor interpretations of radiation tolerance toward a multi-factor perspective, where radiation resistance is integrated with other stressors such as desiccation, oxidative stress, and energy limitation. These combined pressures shape microbial survival strategies through coordinated physiological, molecular, and community-level processes.

Extreme terrestrial environments, including deserts, deep subsurface systems, and space-exposed habitats, provide valuable analogues for astrobiology. These systems demonstrate that life can persist under chronic radiation when supported by protective niches, energy sources such as radiolysis, and ecological interactions. However, differences in environmental complexity and radiation regimes require cautious extrapolation to extraterrestrial settings.

Beyond fundamental insights, radiation-resistant microorganisms hold strong potential for applications in biotechnology and space exploration. These include the development of radiation-resistant biomaterials, bioregenerative life-support systems, and bioremediation strategies, alongside challenges related to planetary protection. Future research should prioritize integrative approaches combining multi-omics, experimental simulations, and predictive modeling to better define the limits of habitability and support upcoming missions to Mars and icy moons.

## Data Availability

No datasets were generated or analysed during the current study.
